# Analysis of oligonucleotide array experiments with repeated measures using mixed models

**DOI:** 10.1186/1471-2105-5-209

**Published:** 2004-12-30

**Authors:** Hao Li, Constance L Wood, Thomas V Getchell, Marilyn L Getchell, Arnold J Stromberg

**Affiliations:** 1 Department of Statistics, 815 Patterson Office Tower, University of Kentucky, Lexington, Kentucky 40506-0027, USA; 2 Department of Physiology, University of Kentucky, 800 Rose Street, Lexington, KY 40536-0298, USA; 3 Department of Anatomy and Neurobiology, College of Medicine, Univeristy of Kentucky, Lexington, KY 40536-0298, USA; 4 309 Sanders-Brown Center on Aging, 800 South Limestone Street, University of Kentucky College of Medicine, Lexington, KY 40536-0230, USA

## Abstract

**Background:**

Two or more factor mixed factorial experiments are becoming increasingly common in microarray data analysis. In this case study, the two factors are presence (Patients with Alzheimer's disease) or absence (Control) of the disease, and brain regions including olfactory bulb (OB) or cerebellum (CER). In the design considered in this manuscript, OB and CER are repeated measurements from the same subject and, hence, are correlated. It is critical to identify sources of variability in the analysis of oligonucleotide array experiments with repeated measures and correlations among data points have to be considered. In addition, multiple testing problems are more complicated in experiments with multi-level treatments or treatment combinations.

**Results:**

In this study we adopted a linear mixed model to analyze oligonucleotide array experiments with repeated measures. We first construct a generalized F test to select differentially expressed genes. The Benjamini and Hochberg (BH) procedure of controlling false discovery rate (FDR) at 5% was applied to the P values of the generalized F test. For those genes with significant generalized F test, we then categorize them based on whether the interaction terms were significant or not at the *α*-level (*α*_*new *_= 0.0033) determined by the FDR procedure. Since simple effects may be examined for the genes with significant interaction effect, we adopt the protected Fisher's least significant difference test (LSD) procedure at the level of *α*_*new *_to control the family-wise error rate (FWER) for each gene examined.

**Conclusions:**

A linear mixed model is appropriate for analysis of oligonucleotide array experiments with repeated measures. We constructed a generalized F test to select differentially expressed genes, and then applied a specific sequence of tests to identify factorial effects. This sequence of tests applied was designed to control for gene based FWER.

## Background

Experiments in which subjects are assigned randomly to levels of a treatment factor (or treatment combinations of more than one factor) and then are measured for trends at several sampling times, spaces or regions (within-subject factors) are increasingly common in clinical and medical research. The analysis of interaction, main effects and simple effects are appropriate for analyzing these types of experiments [1]. Main effects are average effects of a factor, and interaction effects measure differences between the effects of one factor at different levels of the other factor. As an example, this paper studies a 2 × 2 factorial treatment design, in which effects of two factors (treatment and region, for example) are studied and each factor has only two levels (with or without certain treatment, two different regions of studied subjects). The measurements from different regions of a subject are repeated measures on the individual and are correlated. In combination with microarray technology [2], this type of design allows one to investigate how treatments alter changes in gene expression in time or region simultaneously across a large number of genes. Two issues are crucial in the analysis of microarray experiments with repeated measures. Firstly, sources of variability must be identified, and the correlation structure among within-subject measurements needs to be taken into account; and secondly, multiple testing is also an immediate concern if tests of interaction, main effects, and/or simple effects are performed for each gene.

It has been shown that replication is the key not only to increasing the precision of estimation but also to estimating errors associated with tests of significance [3]. Previously, a number of ways to identify and model various sources of errors were proposed for replicated microarray experiments, and corresponding methods of extracting differentially expressed genes were suggested [4-8]. Recently, a linear modelling approach [9] and analysis of microarray experiments using mixed models were also introduced [10-12], in which the dependency structure of repeated measurements at the probe level were discussed. Statistical methods to analyze more complicated experiments, where correlated measurements are taken on one or more factor levels have not yet been fully described. In this study, we modified the two-staged linear mixed models [10], and extended them to more complicated designs.

Attention to the multiplicity problem in gene expression analysis has been increasing. Numerous methods are available for controlling the family-wise type I error rate (FWER) [13-17]. Since microarray experiments are frequently exploratory in nature and the sample sizes are usually small, Benjamini and Hochberg [18]suggested a potentially more powerful procedure, the false discovery rate (FDR), to control the proportion of errors among the identified differentially expressed genes. A number of studies for controlling FDR have followed [17,19-25]. However, these approaches for dealing with the multiplicity problems in microarray experiments are largely focused on relatively simple one-way layout experimental designs, and the number of genes that are involved in an experiment was the major concern. More complicated designs, such factorial designs with two or more factors, intensify the multiplicity problem not only because thousands of genes are involved in an experiment, but also because tests for interactions, main effects, and, possibly, simple effects need to be performed to further characterize differences for each gene. It has not been suggested explicitly, however, how to deal with such multiple-testing problems for two factors (or more than two factors) factorial experiments in the microarray literature.

In this paper, we present a method for analyzing oligonuleotide array experiments with repeated measures using a linear mixed model, which allows us to model variance-covariance structures associated with such complicated experiments. Our method is also related to that of Wolfinger *et al*., 2001, Chu *et al*., 2002, Kerr *et al*., 2000, and Wernisch *et al*., 2002 [5,9-11]. In addition, we construct a generalized F test to test the null hypothesis that all the means for all Disease by Region combinations are equal. Benjamini and Hochberg (BH) procedure of controlling FDR at 5% is used for comparing P values of the generalized F tests. The test to determine whether the interaction term is significant is performed only for each gene with a significant generalized F test. In addition, simple effects are examined for the genes with significant interaction effect and main effects are tested for those differentially expressed genes which do no exhibit significant interaction. In the 2 × 2 factorial, this sequence of tests controls the maximum FWER and, hence the FWER for all genes. We also illustrate how to summarize and categorize the interactions using simple diagrams. We demonstrate our method on the analysis of microarray data from two regions of the brain, the olfactory bulb (OB) and the cerebellum (CER), from control subjects and patients with AD. Although a 2 × 2 experiment was used in this manuscript, our methods can be extended to designs with more than 2 factors or more than 2 levels in one or more factors. The OB was used because AD patients show pronounced decrements in their olfactory sensitivity early in the clinical course of the disease [26]. The cerebellum was selected as a control tissue because it is generally considered to be minimally affected in AD.

## Results

### Analysis of gene expression in OB and CER of controls and AD patients

Based on the statistical methods described (see Methods), 708 genes were considered to be significant by the procedure of controlling FDR at 5% for multiple testing across genes. The largest P-value considered to be significant was 0.0033 determined by the FDR procedure. Among the 708 genes, 137 show significant interaction at the level of 0.0033, 49 genes with significant disease effect (32 were up-regulated and 17 were down-regulated in AD patients) and 559 genes with significant regional effects (331 were up-regulated and 228 were down-regulated in the OB) (Table 1). There were 37 genes that appear on both lists of significant disease and regional effects (not shown). Further validation studies, such as real time RT-PCR, could be performed to examine which interpretation is more reasonable.

**Table 1:** Summary of genes with main effects

**Main effects**	**Disease**		**Region**	
Direction	**I**	**D**	**I**	**D**
# of genes	32	17	331	228
Fold change	1.1~2.9	1.2~2.8	1.1~104.3	1.1~121.7

I: significant upregulation of gene expression;

D: significant downregulation of gene expression.

**Figure 1:**
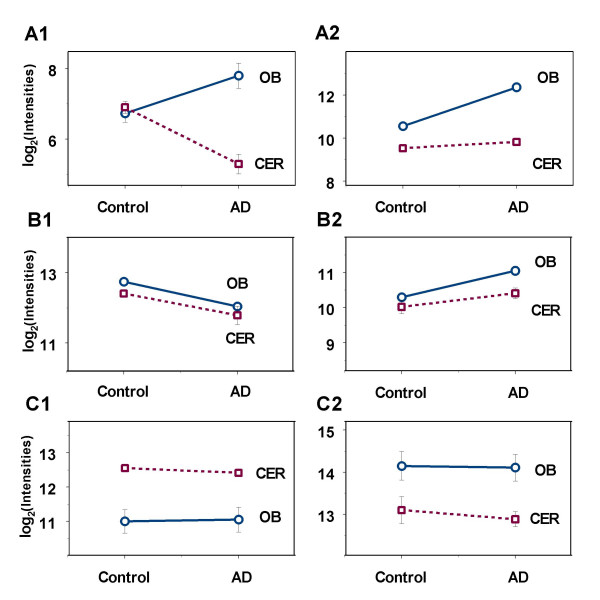
**Simple diagrams to illustrate significant interaction, main effects of Disease and Region**. The average log transformed intensities under control and AD conditions for both OB and CER were plotted together for each gene with either significant interaction or main effects. The two points from each region were connected using a straight line and the non-parallel lines imply interaction. Two examples genes with interaction effect were shown in A. A1, represents a directional interaction and A2 indicates an interaction in magnitude. Two genes with only main effect of disease were illustrated in B, one of which showed down-regulation in AD (B1), while the other genes were upregulated in AD for both OB and CER. In the bottom panel, two genes with only regional differences were shown. The gene in C1 has high expression level in CER, and the gene in C2 has an opposite situation. See also Table 4, 5.

A significant interaction effect for a gene has to be explained so that the gene can be further categorized based on the nature of the possible alterations of their expression levels. The interaction patterns were identified based on the change directions and test results for the following simple effects: control *vs *AD for OB, control *vs *AD for CER, CER *vs *OB for control, and CER *vs *OB for AD. The interaction effects can also be illustrated using simple diagrams by plotting together the average log2 based intensities under control and AD conditions for both OB and CER. Nonparallel lines in a diagram often imply an interaction effect. An interaction effect can be either directional or magnitudinal. In this study, directional interactions refer to the situations when the changes (in gene expression) between AD and control in OB are in the opposite directions compared to the changes between AD and control in CER. In a magnitudinal interaction, the directions of the changes between AD and control are the same but the magnitudes of changes are significantly different. The gene LOC91614 (UniGene Cluster Hs.180545), which encodes novel 58.3 kDa protein, is an example with directional interaction effect. As shown in Figure 1 A1, it is significantly up-regulated 2.08 fold in the OBs of AD patients (Table 2) and significantly down-regulated 3 fold (1/0.33) in their CERs (Table 2). The function of this gene is unknown, but, based on the domains identified in its protein sequence, it is likely to be involved in intracellular signalling cascades. Given this divergence in the direction of regulation in these 2 brain regions, this gene would be of interest for further characterization. The gene encoding the proteolytic lysosomal enzyme cathepsin H (UniGene Cluster Hs.114931) has a different pattern of interaction effects as shown in Figure 1 A2. It was significantly up-regulated 3.51 fold in the OBs of AD patients (Table 2) and shows a slight non-significant trend toward up-regulation in their CERs (Table 2). This is consistent with the pronounced activation of lysosomal enzymes that occurs in regions of the AD brain vulnerable to neurodegeneration (Nixon *et al*., 2000), and with the slight increase in lysosomal density in the CER compared with the pronounced increase in sites of the AD brain with significant neuropathology (prefrontal cortex and hippocampus; [27]). The patterns for other genes with significant interaction effects were also determined by the similar method described above.

**Table 2:** Example of genes with significant interaction effects

**Gene**		LOC91614						Cathepsin H					
		OB			CER			OB			CER		
Con		7.02	6.61	6.58	6.78	7.09	6.83	10.53	10.63	10.49	9.46	9.71	9.42
		6.74 ± 0.25			6.90 ± 0.17			10.55 ± 0.07			9.53 ± 0.16		
AD		7.58	7.59	8.21	5.30	5.03	5.58	12.30	12.41	12.37	9.54	9.82	9.79
		7.80 ± 0.36			5.30 ± 0.28			12.36 ± 0.06			9.72 ± 0.15		
Overall P		0.00058						2.12e-06					
Interaction P		0.00036						5.86e-06					
ConOB *vs *ADOB	Re	+						+					
	fold	2.08						3.51					
	dir	**I**						**I**					
ConCER *vs *ADCER	Re	+						+					
	fold	0.33						1.14					
	dir	**D**						N					
ConOB *vs *ConCER	Re	-						+					
	fold	0.90						2.03					
	dir	N						**I**					
ADOB *vs *ADCER	Re	+						+					
	fold	5.66						6.23					
	dir	**I**						**I**					

ConOB_ADOB: control vs AD for OB; ConCER_ADCER: control vs AD for CER; ConOB_ConCER: OB vs CER for control subjects; ADOB_ADCER: OB vs CER for AD patients. Overall P: the P value of the generalized F test. Interaction P: the P value of the interaction term. Re: the results of the protected Fisher's LSD procedure, where "+" indicates a significant difference and "-" implies a non significant difference ; dir: the direction of alteration in gene expression levels; D for decrease, I for increase and N for no change in AD when comparing control vs AD, or in OB when comparing CER vs OB; fold: fold change of each pairwise comparison calculated from the inverse transformed log2 based data. The fold change of LOC91614 gene expression between AD and control in OB was calculated as: 2^(7.58+7.59+8.21)/3^/2^(7.02+6.61+6.58)/3 ^= 2.08. The fold changes in other situations were calculated in a similar way. Similar notations were also used in Table 3. See also Figure 1.

In the absence of interaction effects, main effects are often meaningful. The genes that have either significant main effect of Disease or Region were also identified and characterized by examining the average difference between AD and controls or the average difference between OB and CER. Main effects can also be illustrated by the simple diagrams described above, in which the lines are often parallel. Four genes were used as examples to illustrate main effect of Disease and main effect of Region (Table 3, Figure 1).

**Table 3:** Example of genes with significant main effects

**Gene**		Log2 based data						Overall P-value	Interaction P-value	Main effect of Disease			Main effect of Region		
		OB			CER					P value	fold	dir	P value	fold	dir
	Con	12.68	12.73	12.83	12.37	12.34	12.53								
		12.75 ± 0.08			12.41 ± 0.10										
**HMGN2**								0.0003	0.6462	0.0009*	0.63	**D**	0.011^†^	1.24	N
	AD	12.01	11.94	12.16	11.53	12.04	11.81								
		12.04 ± 0.11			11.79 ± 0.26										
	Con	10.44	10.22	10.26	10.09	10.16	9.80								
		10.31 ± 0.12			10.02 ± 0.19										
**TSG101**								0.0002	0.0726	0.0021*	1.51	**I**	0.056	1.42	N
	AD	11.20	11.01	10.96	10.30	10.59	10.34								
		11.06 ± 0.13			10.41 ± 0.16										
	Con	11.49	10.91	10.83	12.56	12.47	12.62								
		11.08 ± 0.36			12.55 ± 0.08										
**RELN**								0.0025	0.8328	0.5004	0.92	N	0.0005*	0.37	**D**
	AD	11.33	11.00	10.66	12.45	12.35	12.41								
		11.00 ± 0.34			12.41 ± 0.06										
	Con	14.01	14.54	13.89	13.21	13.34	12.74								
		14.15 ± 0.35			13.10 ± 0.32										
**B2M**								0.0011	0.3767	0.5986	0.94	N	0.0002*	2.20	**I**
	AD	14.03	13.84	14.47	12.70	12.88	13.06								
		14.11 ± 0.32			12.88 ± 0.18										

* indicates the P values pass the FDR 5% criteria. ^† ^indicates the P values are smaller than 0.05 but larger than the critical value 0.0033 determined by the FDR procedure. Overall P-value and interaction P-value are the same with the Overall P and Interaction P in Table 4. P values of main effect of disease and region are the P values of Type III ANOVA test using proc mixed procedure in SAS. Fold of main effect of disease: the ratio of the average intensities of AD (average over OB and CER) over Control (average over OB and CER). Fold of main effect of region: the ratio of the average intensities of OB (average over Control and AD) over CER (average over Control and AD). See also Figure 1.

The genes *HMGN2 *and *TSG101 *both have significant effect of Disease (Table 3). *HMGN2 *(high mobility group nucleosomal binding protein 2) was significantly down-regulated (1.6 fold; *p *= 0.0009) in the OBs of AD patients compared to elderly non-demented controls; there was no significant difference in mean expression levels in the OB and CER as shown in Figure 1 B1 and B2. Its down-regulation in the OBs of AD patients is consistent with the generally reduced level of gene expression that has been described in the AD brain [28]. The gene *TSG101*, was up-regulated 1.51 fold (*p *= 0.0021), with no significant differences in expression levels in the OB and CER. The encoded protein is a member of the mammalian class E vps proteins, which mediate ubiquitination-dependent receptor sorting within the endosomal pathway. The up-regulation of *TSG101 *suggests a potential disruption of OB neurogenesis.

Two examples of genes with Regional effects are *RELN *and *B2M *(Table 3). *RELN *is expressed at lower levels in the OB than in the CER (2.7 fold, *p *<0.0005) as shown in Figure 1 C1 and C2. The encoded protein is a secreted extracellular matrix molecule that interacts with integrin signalling to generate a signal for migratory developing neurons to stop and form layers; thus, a defect in this gene results in improper development of the cerebellum as well as other brain regions [29]. *B2M*, the gene encoding *β*_2 _microglobulin, is expressed at 2.2-fold higher levels in the OB than in the CER (*p *< 0.0002). One potential explanation for the higher levels of *B2M *expression in the OB than the CER is that antigens can enter the brain directly along the pathway provided by the axon of the olfactory receptor neuron or within the sheath of the olfactory nerve; numerous proteins and pathogens enter the brain via this route (*e.g.*, [30,31]. The potentially higher level of antigenic stimulation in the OB may result in the up-regulation of *B2M *expression, which would not occur in the CER due to the lack of such a direct connection with the external environment.

The remaining genes with either significant effects of Disease or Region were also identified and categorized in a similar way and summarized in Table 1.

## Discussion

In this study, we adopted a linear mixed model to analyze oligonuleotide array experiments with repeated measures. We constructed a generalized F test to select differentially expressed genes and compared our method to another frequently used approach. Using the method described above, we identified 708 differentially expressed genes, 137 of which have significant interaction, and 571 genes have main effect of either Disease or Region. Using simple diagrams, we can illustrate and further categorize the interactions and main effects.

This linear mixed model approach allows us to identify various sources of variability, including experimental effects, random effects of subjects and random error. The performance of the generalized F statistic depends on the validity of the assumed covariance structures and the degree of replication. We assumed homoscedastic variances for each gene. This may not be true for all genes in reality. With small sample sizes, which are common in microarray studies, simpler covariance structures which require the estimation of fewer variance components are preferred. Simulation studies showed that, with sample size of 3, the generalized F test performs reasonably well in cases with homoscedastic variances.

We also tested the factorial effects on the 708 genes which were identified by BH procedure using the more conservative Bonferroni adjustment to the *α*-level in order to simultaneously control FDR and the possibility of performing multiple tests for the factorial effects. For example, controlling FDR at 0.05/3 = 1.67%, produced a list of 77 genes. The method we developed is more powerful. In addition, only regional effects were identified without significant interaction and main effect of disease by the alternative method.

In this manuscript, we adopt BH procedure to control FDR at 5% based on the generalized F tests. Any other standard multiple testing procedures may also be applied. A specific sequence of tests was used to identify factorial effects and control the gene-based FWER in our study. For researchers who are interested in all pairwise comparisons among treatment groups, Hayter's modification of the LSD method [32] controls the FWER for all genes.

We also assumed independence of the significant tests among genes. This assumption, which is also adopted in the majority of the microarray literature, may not be completely valid since gene expression is tightly regulated. The correlation among the genes varies from developmental stages, tissue to tissue, etc., and we may never be able to quantify it precisely. The assumption that genes are correlated in small clusters has been adopted by Benjamini and Yekutieli [21] in their FDR control study. This assumption, however, has not been completely verified.

## Conclusions

A linear mixed model is appropriate for analysis of oligonucleotide array experiments with repeated measures, allowing us to quantify various sources of error. We constructed a generalized F test to select differentially expressed genes, and then applied a specific sequence of tests to identify factorial effects. This sequence of tests applied was designed to control for gene based FWER. Our methods can be extended to designs with more than 2 factors or more than 2 levels in one or more factors. The generalized F test can be constructed for any number of factors or levels of factors.

## Methods

### Sources and processing of tissue

OBs were obtained with appropriate informed consent from patients with Alzheimer's disease (AD) and control subjects enrolled in the Biologically Resilient Adults in Neurological Studies (BRAiNS) project of the Sanders-Brown Center on Aging. At autopsy, OBs and pieces of the lateral tip of the cerebellums were removed from 6 females, 3 with AD (mean age, 79.0 years; mean postmortem interval 3.8 h) and 3 controls (mean age, 78.6 years; mean postmortem interval 2.9 h), and immediately placed in liquid nitrogen. BRAiNS control subjects had no clinical evidence of dementia or other neurological problems and scored within the normal range on yearly mental status tests; on neuropathological examination, their brains exhibited age-related but not disease-related changes. AD patients received a diagnosis of probable AD in the Memory Disorders Clinic; on neuropathological examination, their brains met multiple criteria for definite AD and exhibited no indications of complications from cerebrovascular disease [33].

OBs and cerebellum were homogenized in TRI-Reagent (Molecular Research, Inc., Cincinnati, OH), and total RNA was extracted according to the manufacturer's protocol. RNA concentration was determined spectrophotometrically; its integrity and quality were assessed by spectrophotometry, agarose gel electrophoresis, and Bioanalyzer (Agilent, Technologies, Wilmington, DE) virtual gels. Following target preparation, the samples were hybridized onto the Affymetrix Human Genome U133_A and _B GeneChips at the University of Kentucky Microarray Core Facility according to Affymetrix protocols.

### Experimental design

OBs and pieces of the lateral tip of the cerebellums were previously removed from each of 3 control subjects and 3 patients with AD (all female with similar ages). Total RNA was extracted from OB and CER tissues for each subject. Five *μ*g RNA from the OB and CER of each individual were hybridized with Affymetrix Human Genome U133_A and _B chips (2 GeneChips/tissue/individual = 24 GeneChips). Data from U133_A and _B chips for each RNA sample were combined to give 12 data sets with signal intensities for 44828 targets. Under the assumption of independence among genes, we have a 2 × 2 factorial design for each gene with one factor being either control or AD and with repeated measures (regions, OB or CER) on each subject. The arrangement for the 2 × 2 mixed factorial design in this experiment is shown as in Table 4, where *μ_11_*, *μ_12_*, *μ_21_*, and *μ_22 _*denotes the average log 2 based expression levels measured in OB of controls, CER of controls, OB of AD patients and CER of AD patients respectively. Corresponding measurements from the same subject are correlated and they are marked as same color. Our primary interests are to identify various sources of variability and differentially expressed genes.

**Table 4:** The arrangement for the 2 × 2 factorial design with repeated measures

	**OB**	**CER**
	
**Control**	***μ_11_***	***μ_12_***
**AD**	***μ_21_***	***μ_22_***

***μ_11_***, ***μ_12_***, ***μ_21_***, and ***μ_22 _***are the true means of measurements in OB of controls, CER of controls, OB of AD patients and CER of AD patients respectively.

### Data preparation

#### Normalization

Background correction and initial total intensity normalization were first performed for the microarray raw data using Affymetrix Version 5 software [34], resulting in gene intensities for each gene-chip combination. The log intensities values were used in later processing. We chose the local regression method (loess) [35-37] to normalize the chips within each of the four treatment combinations. The total intensity method was performed to normalize array across treatment combinations.

#### Data Filtering

In our study, all positive control genes and genes that resulted in an "absent" call for all chips were removed from further analysis. If there was no evidence that these genes were expressed in any of the samples, then these genes can be removed to reduce problems associated with multiple comparisons. Other methods of removing low intensity points were also suggested by Bolstad *et al*., 2003 [37]. All ESTs were also removed from the analysis. Since the primary interest of these experiments is to identify known genes that are differentially regulated, eliminating ESTs will further reduce problems with multiple comparisons. After data filtering steps, 10,590 genes remained, and the base-2 logarithms of background-corrected and normalized intensities of these genes were subject to further statistical analyses.

### Algorithm and analysis

#### Analysis of variance components

We use a linear mixed model to describe the experiment.

Let Y_*gijk *_be the base-2 logarithm of background-corrected and normalized intensity of the g*th *gene, *g *= 1, ..., 10590, in the i*th *Treatment group *i *= 1, 2, from the j*th *Region, *j *= 1, 2, on the k*th *subject *k *= 1, 2, 3. "Treatment" here signifies the health condition of the subjects (controls or AD patients). A complete linear mixed model for this experiment:

Y_*gijk *_= *μ *+ D_i _+ *S*_*ik *_+ R_j _+ (DR)_ij _+ *A*_*ijk *_+ G_g _+ (GD)_gi _+ (GR)_gj _+ (GDR)_gij _+ *ε*_*gijk*_,     (1)

where *μ *is the grand mean, D_i _and R_j _and are the main effects of treatments, regions respectively, and (DR)_ij _are the treatment-region interaction effects. Here *S*_*ik *_are the random effects of subjects within disease group and *A*_*ijk *_are the random effects of chips. The symbols G_g_, (GD)_gi_, (GR)_gj _and (GDR)_gij _represent the main effect of gene, gene-treatment interaction effects, gene-region interaction effects, gene-treatment-region interaction effects, while *ε*_*gijk *_are the additive stochastic errors

In general, it is impractical, using currently available software, to fit linear models such as (1) with microarray data involving manipulation of the full covariance matrix of observation variables that usually contains thousands of levels. To be conceptually and computationally more efficient, Wolfinger *et al*., 2001 [10] suggested a two-step model to separate experimental-wise systematic effects (normalization sub-model) and the remaining effects for each gene (gene sub-model). In our case, however, the design matrix for the fixed effects of D_i_, R_j _and (DR)_ij _is orthogonal to the design matrix for the fixed effects involving each gene, including G_g_, (GD)_gi_, (GR)_gj _and (GDR)_gij_. Therefore, the normalization model has no effect on the inference for each gene under the assumption in (1). A simpler model can be adopted for each gene, and the random effect *S*_*ik *_is absorbed by *S*_*gik *_terms and *A*_*ijk *_is absorbed by *ε*_*gijk *_terms. We make standard stochastic assumptions that the random effects *S*_*gik*_, and *ε*_*gijk *_are normally distributed with zero means with variances *σ*_gs_^2^, and *σ*_*g*_^2 ^respectively. These random effects are assumed to be independent both across their indices. The model equation then becomes

Y_*gijk *_= *μ*_g _+ D_gi _+ R_gj _+ (DR)_gij _+ *S*_*gik *_+ *ε*_*gijk*_.     (2)

In matrix notation, the model equation for each gene can be written

**Y **= **X*β ***+ Z**u **+ **ε, **    (3)

where **Y **is a vector of observations, **X **and Z are matrices of known constants for the fixed effects and random effects, respectively, ***β ***is a vector containing fixed effect parameters D_gi_, R_gj_, and (DR)_gij_, **u **is a vector of random effects, and ***ε ***is the error or residual vector. Therefore, **Y **~ MVN (**X*β,V***) where *V *= ***ZDZ' ***+ **Σ**. The covariance matrices ***D ***= var(**u**) and **Σ **= var(***ε***) can have any valid variance-covariance matrix form. The variances of gene specific subject effects *S *can vary for different treatments and different genes, while *ε *effects can have different variances for different treatments, regions and different genes. The remaining terms are fixed effects. All effects and variance components in the model can be estimated using the method of restricted maximum likelihood (REML) [38].

In the homogeneous variance case assumed here, since observations across subjects are independent, the variance-covariance matrix for gene g, ***V_g_***, is block diagonal where

***V_g _***= *diag *(**Σ_*g*_**)

and



If the assumption of homoscedasticity is not viable, the variance-covariance for gene g can easily be accommodated by allowing **Σ_*g *_**to vary across disease groups.

#### Estimation of model parameters

The estimate of primary interest is ***β***, which containing treatment, region effects and treatment-region interaction for each gene. For each gene, ***β ***is estimated by



The estimated  has covariance



where in practice components of ***V ***are replaced by their REML estimates. See Verbeke and Molenberghs (2000) [1] for methods to derive equations (3)–(5) and REML estimates of the random components in details.

#### Construct a generalized F test

Genes showing significant interaction effects are defined as those in which the difference in expression levels between control and AD is not the same with the difference between OB and CER. Main effects are meaningful in the absence of interaction effect. Genes showing a significant disease-related effect or main effect of disease are defined as those either under- or over-expressed by AD patients compared to controls at the same extent in both OB and CER, while genes with significant main effect of region are those either under- or over-expressed in OB compared to CER at the same extent by both AD and controls. If the expression levels for a gene are the same across all treatment-region combinations, then there will be neither significant interaction nor main effects; therefore this gene should be excluded from further analysis. The expression of other genes may be altered by treatment or/and region effects, and further analysis of these genes is needed to characterize the experimental effects. Therefore the first step to select differentially expressed genes in factorial designs is to choose those for each of which the hypothesis of equality of all cell means, *μ_11 _*= *μ_12 _*= *μ_21 _*= *μ_22_*, is rejected. Because of the specific variance-covariance structure for a repeated measures experiment with two levels of the within subject factor, it is convenient to test the equivalent composite hypothesis for each gene g which is stated in terms of the main effects and the interaction. Specifically, we consider



We can test this composite null hypothesis of no interaction and main effects simultaneously by setting up 3 corresponding linear contrasts listed in Table 5. A contrast is a linear combination of parameters, for which the coefficients sum to zero [39]. Let **L **be the 3 × 8 matrix containing the coefficients of the 3 contrasts, then *H_o _*is simplified as **L*β ***= **0**, where **L*β ***is estimable, and can be tested using the generalized F test

**Table 5:** Set up hypothesis using linear contrasts

***H_o_***		**L**							
**Effects**	**Mean**	**D**_*g1*_	**D**_*g2*_	**R**_*g1*_	**R**_*g2*_	(**DR**)_*g11*_	(**DR**)_*g12*_	(**DR**)_*g21*_	(**DR**)_*g22*_

(DR)_*gij *_= 0	(*μ_21 _*- *μ_11_*) - (*μ_22 _*- *μ_12_*) = 0	0	0	0	0	-1	1	1	-1
D_*gi *_= 0	(*μ_11 _*+ *μ_12_*) - (*μ_21 _*+ *μ_22_*) = 0	2	-2	0	0	1	1	-1	-1
R_*gj *_= 0	(*μ_11 _*+ *μ_21_*) - (*μ_12 _*+ *μ_22_*) = 0	0	0	2	-2	1	-1	1	-1

The hypothesis in terms of model parameters and means were listed. The coefficients for the model parameters of the linear contrasts were determined for the corresponding hypotheses.



Under *H_o_*, the generalized F is distributed approximately as Snedecor's F with degrees of freedom rank(L) and *ν *(**F**_[rank(L), *ν*]_). Since the variance-covariance matrix V satisfies a compound symmetry condition, in our example this statistic is distributed as **F**_[3, 4]_. Under other assumptions of the variance-covariance structures, the denominator degrees of freedom *ν *can be approximated by the degrees of freedom to estimate ***L(X'V^-1 ^X)^-1 ^L' ***using Satterthwaite's procedure [38,40]. Details about how to select appropriate covariance structures were discussed by Littell et al. (1996) [38] and Keselman et al. (1998) [41].

#### Adjustment for multiple tests

Multiple testing problems in microarray experiments with factorial designs are at least two-fold. Usually, hypothesis tests are performed for each of thousands of genes involved, and tests of main effects and interactions may also be needed for each gene. Based on the generalized F test we constructed above, we now suggest a method for adjusting multiple tests.

The most commonly used methods to adjust multiple tests are of controlling either FWER or FDR. These methods are first applied to the P-values from the generalized F tests, providing a list of genes that exhibit significant difference among the four cell means of Disease by Region combination. Some of these genes may have significant interactions, or only the main effects of treatment and/or region are significant. Further characterizing the significant interactions are one of the major interests for researchers, and methods for investigate interaction contrasts are available [42-44]. In our study, simple effects were examined for the genes the have a significant interaction to detect the difference between specific comparisons. Protected by the generalized F test, Fisher's least significant difference test (LSD) method can be used to test the necessary simple effects. Here the appropriate error terms for these simple effects depend on whether the comparisons involve measurements from same Disease groups or not. This sequence of tests proposed in this paper are more powerful, while still allowing for the control of FWER or FDR, compared to directly adjusting P-values using BH procedure with Bonferroni correction. In the latter method, if we control overall FDR at 5%, we would perform BH procedure at level of 1.67% or 0.05/3 for each test of interaction, main effect of disease or region.

#### Recipe of the analysis

A short summary of the statistical methods used in this study follows:

1. Linear mixed models were used to describe the data based on the experimental design and some common assumptions, and the variance components were specified.

2. For each gene, a generalized F test was performed based on the described model, and the corresponding P-value was obtained.

3. To adjust the multiple tests for numbers of genes, the BH method of controlling FDR [18] at 5% was applied to the P-values obtained above, providing a list of genes (list I) that exhibit significant differences among the means of the Disease*Region combinations.

4. Using *α*_*new*_, which equals to the largest P-value considered to be significant in step3 as the cut-off point, we choose genes with significant interactions (list II) from list I and, for genes in list II, to test the simple effects. By complete enumerating of all possible combinations of main effects and interaction effect, one can prove that *α*_*new *_is an appropriate choice to control the FWERs while selecting genes with either significant interaction or main effects in 2 × 2 factorial experiments. From the remaining genes, significant main effects of either disease or region (list III) were selected. In the example used in this study, *α*_*new *_= 0.0033.

#### Statistical software

Data normalization and generation of simulated data were performed using S-plus version 6.1. We used SAS (version 9.0) proc mixed procedure to do Model fitting and significance analysis. The SAS program implementing linear mixed models for the AD data is available on request from the first author.

### Simulation studies

**Figure 2:**
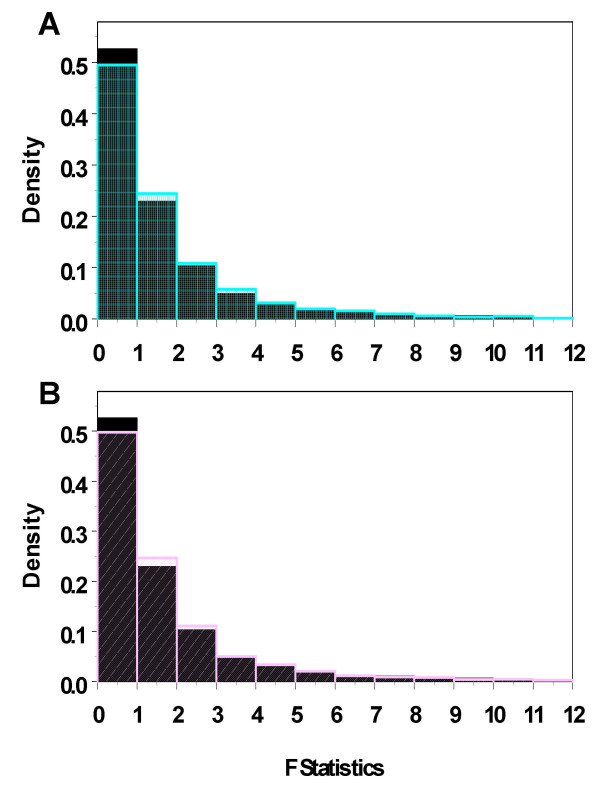
**Histograms of Simulated F statistics**. The histograms of the F statistics from F_[3, 4] _(grey in A, B), simulated data with same covariance structure among individuals (cyan, case I in A) or unequal variance for subjects from controls and AD patients (blue, case II in B). Case I has slightly larger tail than random generated F values, and the right tail of case II were thicker than both of cases above.

We constructed a generalized F test to select differentially expressed genes (see method). To assess the performance of the constructed generalized F test with small sample sizes, we performed simulation studies. Since expression levels of genes in the OB or CER from an individual (either a control or patient with AD) are considered to be repeated measures, correlated data should be generated for the simulations. First, we studied the case (case I) with equal variance and covariance structure for each individual subject (control or AD patient). We generated 10,000 sets of correlated data; each set has 6 bivariate observations, with mean 20 and the following covariance structure for each subject under either Disease condition (i = 1 for control, and i = 2 for AD; j or j' = 1 for OB, and j or j' = 2 for CER, k = 1, 2, 3):



where Y_*gijk *_and Y_*gij'k *_are measurements from the j^*th *^and j'^*th *^levels of Region for the kth subject in the i*th *level of Disease for gene g. The generalized F-statistics were computed for each of the 10,000 data sets and the histogram of the generalized F-statistics was compared with that of randomly generated F values from a **F**_[3, 4] _distribution as shown in figure 2A. The histogram of the generalized F-statistics has a slightly larger tail. The proportion of the generalized F-statistics that were no larger than the critical value, F_[3, 4, *α *= 0.05] _= 6.59 was 5.12%, instead of the nominal 5%, 4.97% random generated F values were smaller than 6.59.

More complicated variance and covariance structure can also be assumed. For example, the controls and AD patients may have a different covariance matrix. We then generated simulated data to study cases like above (case II). Using the same covariance structure as above, we generate 10,000 sets of data for controls. For AD, we generated 10,000 sets of data using a different covariance structure



Then we computed the generalized F-statistics and compared them with randomly generated F values described above (Figure 2B). The histogram of the generalized F-statistics has a slightly larger tail than those of both random generated F values and case II. There was 5.42% of the generalized F-statistics in case II were larger than the critical value 6.59. With small sample size in both cases (n = 3), the constructed generalized F-statistics behave reasonably well.

## Authors' contributions

HL carried out the study. AJS, and CLW supervised the study. TVG and MLG carried out the molecular genetics studies. All authors contributed to the writing of this manuscript. All authors read and approved the final manuscript.
